# Effect of Fillers on the Recovery of Rubber Foam: From Theory to Applications

**DOI:** 10.3390/polym12112745

**Published:** 2020-11-19

**Authors:** Thridsawan Prasopdee, Wirasak Smitthipong

**Affiliations:** 1 Specialized Center of Rubber and Polymer Materials in Agriculture and Industry (RPM), Department of Materials Science, Faculty of Science, Kasetsart University, Chatuchak, Bangkok 10900, Thailand; thridsawan@gmail.com; 2 Office of Research Integration on Target-Based Natural Rubber, National Research Council of Thailand (NRCT), Chatuchak, Bangkok 10900, Thailand; 3 Office of Natural Rubber Research Program, Thailand Science Research and Innovation (TSRI), Chatuchak, Bangkok 10900, Thailand

**Keywords:** rubber foam, filler, charcoal, silica, compression set, recovery, thermodynamics

## Abstract

Natural rubber foam (NRF) can be prepared from concentrated natural latex, providing specific characteristics such as density, compression strength, compression set, and so on, suitable for making shape-memory products. However, many customers require NRF products with a low compression set. This study aims to develop and prepare NRF to investigate its recoverability and other related characteristics by the addition of charcoal and silica fillers. The results showed that increasing filler loading increases physical and mechanical properties. The recoverability of NRF improves as silica increases, contrary to charcoal loading, due to the higher specific surface area of silica. Thermodynamic aspects showed that increasing filler loading increases the compression force (*F*) as well as the proportion of internal energy to the compression force (*F*_u_/*F*). The entropy (*S*) also increases with increasing filler loading, which is favorable for thermodynamic systems. The activation enthalpy (∆*H*_a_) of the NRF with silica is higher than the control NRF, which is due to rubber–filler interactions created within the NRF. A thermodynamic concept of crosslinked rubber foam with filler is proposed. From theory to application, in this study, the NRF has better recoverability with silica loading.

## 1. Introduction

Natural rubber (NR) is a biobased polymer usually applied in the rubber industry to manufacture products such as gloves, pillows, mattresses, tires, and so on. It is derived from the *Hevea brasiliensis* tree as colloidal latex, which presents other substances or nonrubber components, such as proteins, fatty acids, inorganic matters, and so on. This natural latex can be stabilized by the base chemical to maintain prolonged storage [1,2,3,4].

Many products, such as pillows and mattresses, are made from rubber foam prepared from concentrated natural latex, which provides specific characteristics. Rubber foams are generally porous and elastic with ventilated surfaces. These foams are made into lightweight products for comfort applications such as pillows and mattresses [5,6]. From the perspective of mechanical properties, rubber foam can either be soft or firm depending on the formula of the compounded latex. Concentrated natural latex is mixed with chemical agents, consisting of a blowing agent, vulcanizing agent, accelerators, an activator, antioxidants, a gelling agent, and so on, to form compounded latex [1,7]. All the chemical agents have to be approximately ground into the micron scale because they can be easily mixed with the concentrated natural latex since the rubber particle is also present in the micron scale [8].

Rubber foam typically presents shape-memory polymer characteristics, which can be altered by useful properties from the external stimuli [9]. This ability enables responsive materials with form-fitting, actuation, and sensing characteristics in industries such as furniture, biomedicine, aerospace, and so on [10,11]. The viscoelastic properties of rubber foam play an important role in the shape-memory effect. However, many customers require natural rubber foam products that return to their initial form in minimal time or have better recoverability. One can enhance the elasticity of rubber foam to improve the recovery performance of rubber foam products.

In 2015, Bashir et al. [12] used eggshell powder (ESP) as a filler in NRF. The contribution of ESP to an increase in mass increases the density of NRF with increasing ESP loading. At low ESP loading, the tensile strength of the ESP-filled NRF initially drops because the filler is not enough to reinforce the NRF. The tensile strength increases after adding more filler loadings due to the reinforcement effect of ESP. The increment of ESP loading causes the ESP-filled NRF to lose recoverability, caused by nonelastic deformation, indicating the deformation of the hard phase from the increase of ESP loading. Another filler in NRF is rice husk powder (RHP). Ramasamy et al. [13] found that the recovery percentage decreases with the increase of RHP loading, whereas the compression strength is increased with increasing RHP loading.

Many previous works have shown that the recoverability of NRF is decreased with increasing filler loading. The main objective of this study is to enhance the recoverability of NRF; thus, we investigated a crosslinked NRF filled with commercial filler consisting of activated charcoal and silica, which are widely used in the rubber industry. This is a useful channel to better understand the relationship of the mechanical properties and thermodynamic theory of NRF, which can be applied for the recovery of rubber foam products.

## 2. Materials and Methods

### 2.1. Materials

The following materials were used: high-ammonia-concentrated natural latex (Num Rubber and Latex Co., Ltd., Trang, Thailand), potassium oleate (KO: 20% aqueous dispersion prepared by Thanodom Technology Co., Ltd., Bangkok, Thailand), sulfur (S: 50% aqueous dispersion prepared by Thanodom Technology Co., Ltd., Bangkok, Thailand), zinc diethyldithiocarbamate (ZDEC: 50% aqueous dispersion prepared by Thanodom Technology Co., Ltd., Bangkok, Thailand), zinc-2-mercaptobenzothiazole (ZMBT: 50% aqueous dispersion prepared by Thanodom Technology Co., Ltd., Bangkok, Thailand), Wingstay L (WingL: 50% aqueous dispersion prepared by Thanodom Technology Co., Ltd., Bangkok, Thailand), zinc oxide (ZnO: 28% aqueous dispersion prepared by Thanodom Technology Co., Ltd., Bangkok, Thailand), diphenylguanidine (DPG: 28% aqueous dispersion prepared by Thanodom Technology Co., Ltd., Bangkok, Thailand), sodium silicofluoride (SSF: 23% aqueous dispersion prepared by Thanodom Technology Co., Ltd., Bangkok, Thailand), toluene (AR grade, RCI Labscan Co., Ltd., Bangkok, Thailand), bamboo charcoal (BET 2–4 m^2^/g for a 40 μm charcoal particle size, CharcoalHome Co., Ltd., Bangkok, Thailand), precipitated silica (BET 170–190 m^2^/g for a 10–20 nm silica particle size, Extol Technology Co., Ltd., Dongguan, China).

### 2.2. Preparation of Rubber Foams

Rubber foams were prepared in nine types, as presented in Table 1. For each sample, high-ammonia-concentrated natural latex was weighed, and the ammonia was eliminated using a blender at 80 rpm for 1 min. Filler was later added and mixed for 1 min. The speed of the blender was increased to 160 rpm, and KO was subsequently added and mixed for 10 min until the volume of foam increased by approximately three times. The blending speed was reduced to 80 rpm. A group of chemicals (S, ZDEC, and ZMBT as vulcanizing chemicals and WingL) was added, and mixing continued for 1 min. After that, another group of chemicals (ZnO and DPG) was added, and mixing continued for 1 min. SSF was added at last and mixed for 3 min. During the mixing of SSF, gel-forming was repeatedly tested until the gel point was almost reached. The foam was then poured into molds, and the lids were closed afterward. The samples were left at room temperature for 45 min. Finally, the samples were vulcanized in a hot air oven at 90 °C for 2 h, removed from the molds, washed, and dried at 70 °C.

### 2.3. Characterization

Density was measured from the weight of the rubber foam sample and the measured volume of the rubber foam sample, calculated as follows:

(1) $$\mathrm{Density}\;=\;\frac{M}{V}\;\;$$

where *M* is the weight of the rubber foam sample (kg), and *V* is the volume of the rubber foam sample (m^3^).

The crosslinking density of the NRF was determined by the swelling method. Small pieces of the NRF were immersed into toluene to reach the equilibrium of swelling. The crosslinking density of rubber (ν) can be calculated according to the Flory–Rehner equation [14,15,16] as follows:

(2) $$\nu =\;-\frac{1}{V_{s}}\left[\frac{\ln\left(1-V_{r}\right)+V_{r}+\chi V_{r}^{2}}{V_{r}^{\frac{1}{3}}-\frac{V_{r}}{2}}\right]\;\;$$

where *V*_s_ is the molar volume of toluene (106.9 cm^3^/mol), *V*_r_ is the volume fraction of rubber in the swollen network, and χ is the parameter between the rubber and solvent interaction (0.43 + 0.05 *V*_r_). The NRF sample in toluene was kept in the dark at an ambient temperature. It was removed from the toluene and weighed every day until the equilibrium was reached (approximately 7 days). Finally, the sample was dried in an oven at 60 °C for 24 h and weighed again.

The functional group of the NRF sample was determined by attenuated total reflection–Fourier transform infrared spectroscopy (ATR–FTIR VERTEX 70, Bruker, Billerica, MA, USA). The NRF sample was put on a Ge crystal probe at 500–4000 cm^−1^.

Compression stress of the NRF was determined in 3 replications by a texture analyzer (TA.XT Plus, Stable Micro Systems, Godalming, Surrey, UK) using a platen probe with a diameter of 100 mm and a speed of 0.1 mm/s, adapted from ISO 3386. The NRF sample was 45 mm width × 45 mm length × 21.5 mm thickness, compressed at 75% from the foam surface at room temperature.

The compression set was conducted according to ISO 1856 Method C by measuring the height of the sample (*d*_o_). The sample was then compressed at 75 ± 4%height for 72 h at an ambient temperature. Finally, the sample was released from the compression for 30 min, the height of the sample was measured again (*d*_r_), and %compression set was calculated as follows:

(3) $$\%\mathrm{compression}\;\mathrm{set}\;=\;\left[\frac{d_{o}-d_{r}}{d_{o}}\;\times \;100\right]\;\;$$

Furthermore, %recovery of rubber foam can be calculated as follows:

%recovery = 100 − %compression set(4)

The morphological properties of the NRF sample were characterized by scanning electron microscopy (SEM) analysis (FEI, Quanta 450 FEI, Eindhoven, Netherlands). The NRF sample was cut into small pieces and coated with gold in a sputter coater (Polaron Range SC7620, Quorum Technologies Ltd., Kent, UK).

For the thermodynamic aspects, the NRF was determined in 3 replications by a texture analyzer (TA.XT Plus, Stable Micro Systems, Godalming, Surrey, UK) using a platen probe with a diameter of 100 mm and speed of 0.1 mm/s. The sample was 45 mm width × 45 mm length × 21.5 mm thickness and prepared at the test temperature 10 min before starting the experiment. The sample was then compressed from 20% strain to 70% strain from the original foam shape at various temperatures (25, 35, 45, 55, and 65 °C). After that, the force–temperature relationship graph was plotted, and the obtained variables were used to calculate the compression force (*F*) due to the changes of internal energy (*F*_u_) and entropy (*F*_s_) associated with the deformation process.

Other thermodynamic aspects were studied by a temperature sweep of the NRF sample using dynamic mechanical analysis (DMA1, Mettler Toledo, Columbus, OH, USA). The NRF sample was cut into a sample of 7 mm width × 7 mm length × 7 mm thickness and tested at temperatures from 80 to 80 °C. The changes of Gibbs free energy (Δ*G*) and entropy (Δ*S*) were calculated. The activation enthalpy of the transition process ((Δ*H*_a_)_avg_) for the relaxation of the backbone motion of rubber chains is related to the area under the tan δ peak, which can be calculated by [17]:

(5) $$(\Delta H_{a}{)}_{\mathrm{avg}}\;=\;\frac{\left(\ln E_{g}-\ln E_{r}\right)\pi RT_{g}^{2}}{t_{A}}\;\;$$

where *t*_A_ is the area under the tan δ peak, *E*_g_ is the storage modulus at the glassy state, *E*_r_ is the storage modulus at the rubbery state, *R* is the gas constant (8.3145 J/mol·K), and *T*_g_ is the glass transition temperature (K) of the NRF.

## 3. Results and Discussion

### 3.1. Physical and Chemical Properties

**Figure 1:**
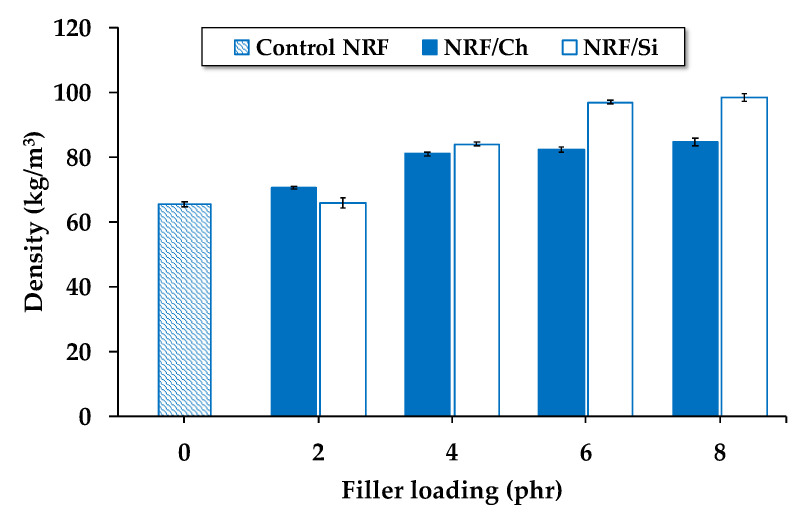
Effect of filler loading on the density of the NRF samples.

The density of the NRF samples increases with increasing filler loading, as presented in Figure 1. This is due to increasing filler loading, which causes an increase in the mass of the NRF with filler. Kudori and Ismail [18] found that foam density increases as the filler size decreases. The smaller filler size hinders pore formation and increases the continuous matrix amount. In the present study, silica filler (nanometer) is smaller than charcoal (micrometer). Therefore, NRF with silica loading exhibits a higher density than NRF with charcoal loading at a given filler concentration. Increasing charcoal loading barely affects the density, which may be because charcoal acts as a nucleating agent during the process of foam growth [19,20,21].

**Figure 2:**
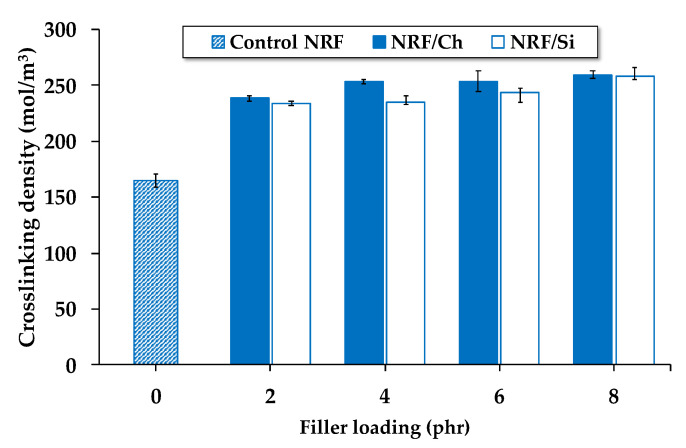
Effect of filler loading on the crosslinking density of the NRF samples.

As presented in Figure 2, the crosslinking density of the NRF samples increases with the presence of filler, which may be due to the additional carbon–sulfur linkages formed by the chemical reaction between the rubber and filler [22]. Another reason is that the amplification of the deformation of rubber chains in the NRF with filler loading is more than the control NRF. Fillers in NRF extend rubber chains due to the interaction of rubber chains at the filler surface, i.e., some rubber chains may be occluded in the voids of the filler, causing the extension of rubber chains and leading to an increased crosslinking density. However, at the same loading of vulcanizing chemicals, increasing filler loading causes fewer differences in the crosslinking density.

**Figure 3:**
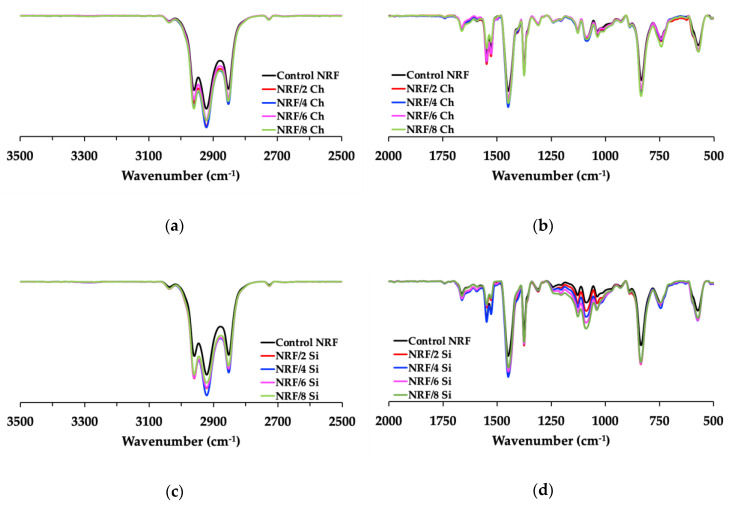
ATR–FTIR spectra of the NRF samples: (**a**,**b**) NRF with charcoal loading; (**c**,**d**) NRF with silica loading.

The chemical compositions of the control NRF and NRF with fillers were analyzed by ATR–FTIR (Figure 3). There is no significant difference in the functional groups of NRF [7,23]. There is almost no difference for the NRF with charcoal loading due to carbonization at high temperatures, which causes the charcoal powder to exhibit a hydrophobic nature [24]. However, there is a band growing at 1100 cm^−1^ for the NRF filled with silica. This band corresponds to the vibration absorption of the silane group (Si–O–C) [25], present in the rubber network, which usually exhibits within the ranges 800–850 and 1100–1200 cm^−1^.

### 3.2. Mechanical and Morphological Properties

**Figure 4:**
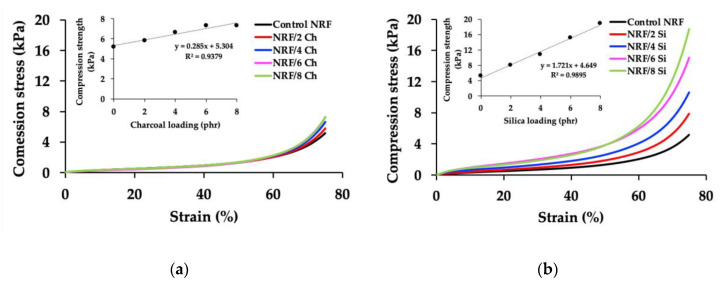
Compression stress of the NRFs: (**a**) NRF with charcoal loading; (**b**) NRF with silica loading.

The control NRF and NRF with fillers were compressed up to 75% (Figure 4). The compression strength at maximum compression shows that the NRF with silica loading has higher compression strength than the NRF with charcoal loading at a given filler concentration. There are two different regions in the compression stress–strain curves of foam materials: elasticity at the low-strain region and solidity at the high-strain region [7]. Increasing filler loading increases the solidity or stiffness of the NRF at high strain, where the foam cells with each other, leading to the immediate increase of compression stress. There is also the stress-induced crystallization of rubber chains that affects the increase of compression stress at high strain. The addition of more than 2 phr of charcoal causes the foam to be sticky, explaining the unfavorable interaction within the foam structure. Although the crosslinking density of the NRF with various silica loadings is almost identical, the compression strength is significantly different. The better interaction within the foam structure is due to the smaller silica filler size, which has more specific surface areas compared to the charcoal filler. We found a linear relationship between compression strength and filler loading in both types of fillers. This relationship depends on the rubber–filler interaction, presented as:

*C*_Ch_ = 0.285⋅[Ch] + 5.304 (6)

*C*_Si_ = 1.721⋅[Si] + 4.649 (7)

where *C*_Ch_ is the compression strength of the NRF with charcoal loading (kPa), *C*_Si_ is the compression strength of the NRF with silica loading (kPa), [Ch] is the concentration of charcoal filler (phr), and [Si] is the concentration of the silica filler (phr).

**Figure 5:**
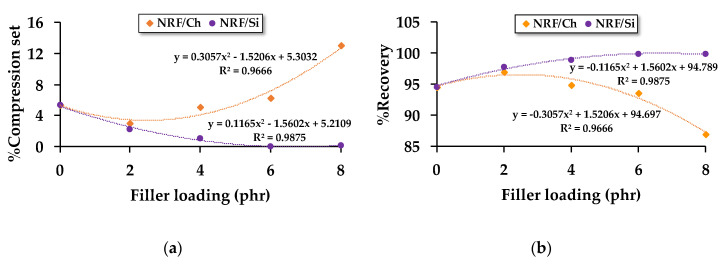
Compression recovery properties of the NRFs: (**a**) %compression set of the NRF with different filler loadings; (**b**) %recovery of the NRF with different filler loadings.

The compression set describes the elastic behavior of the NRF, which relates to the material’s recovery percentage. Figure 5 shows that increasing charcoal loading increases the compression set percentage and decreases the recovery percentage. As mentioned above, the addition of more than 2 phr of charcoal causes the foam to be sticky. Thus, when the NRF with more than 2 phr of charcoal loading is compressed at 75%height of its thickness for a long period (72 h), the ability to return to its original shape is decreased. On the contrary, increasing silica loading decreases the compression set percentage and increases the recovery percentage. Decreasing the compression set percentage indicates higher elasticity. Hence, the NRF with silica loading possesses higher elasticity than the NRF with charcoal loading. Microsized charcoal has been shown to behave like eggshell powder and rice husk powder in previous works [12,13], which decreased the percentage of NRF recovery when filler loading is increased and vice versa with NRF-filled nanosized silica. Therefore, we can propose the relationship between recoverability of NRF and filler concentration as the following polynomial equation:

%*R*_ch_ = −0.3057⋅[Ch]^2^ + 1.5206⋅[Ch] + 94.697 (8)

%*R*_Si_ = −0.1165⋅[Si]^2^ + 1.5602⋅[Si] + 94.789 (9)

where %*R*_Ch_ is the recoverability of the NRF with charcoal loading (%), %*R*_Si_ is the recoverability of the NRF with silica loading (%), [Ch] is the concentration of charcoal filler (phr), and [Si] is the concentration of silica filler (phr).

**Figure 6:**
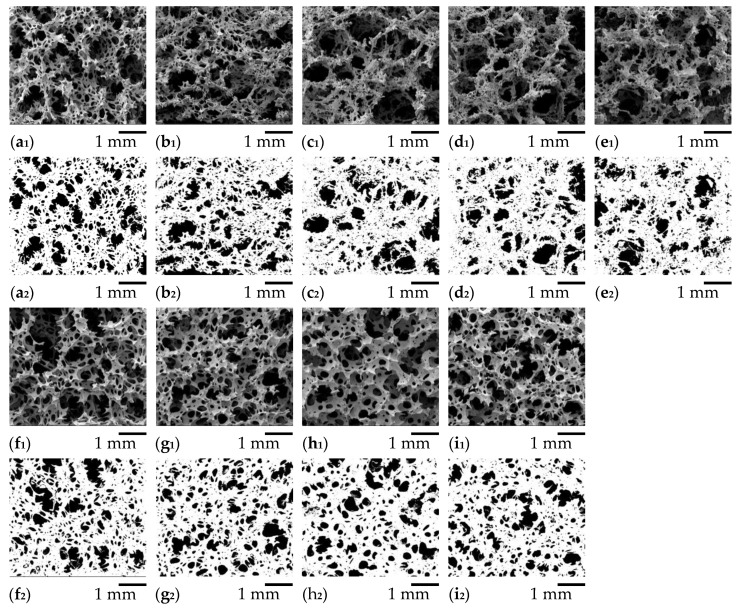
SEM images of: (**a1**) control NRF; (**b1**) NRF/2 Ch; (**c1**) NRF/4 Ch; (**d1**) NRF/6 Ch; (**e1**) NRF/8 Ch; (**f1**) NRF/2 Si; (**g1**) NRF/4 Si; (**h1**) NRF/6 Si; (**i1**) NRF/8 Si. The SEM images with ImageJ analysis of: (**a2**) control NRF; (**b2**) NRF/2 Ch; (**c2**) NRF/4 Ch; (**d2**) NRF/6 Ch; (**e2**) NRF/8 Ch; (**f2**) NRF/2 Si; (**g2**) NRF/4 Si; (**h2**) NRF/6 Si; (**i2**) NRF/8 Si.

The morphological properties of NRF were investigated by SEM. The micrographs indicated that all types of NRF contain a cellular structure that exhibits an interconnected network of open cells, as presented in Figure 6. Porosity analysis was determined by the ImageJ software by adjusting the threshold of the images. The white region corresponds to the pore shape, whereas the dark region corresponds to the open holes or pores (Figure 6). The interconnected porosity of these NRFs is an important parameter that affects the mechanical properties [7]. This result can be explained by the cell density value. The cell density (*d*_cell_) is calculated as follows [26]:

(10) $$d_{\mathrm{cell}}\;=\;\frac{3}{4\pi r^{3}}\left(1-\frac{\rho }{\rho_{s}}\right)\;\;$$

where ρ is the foam density, $\rho_{s}$ is the solid phase density (NR 0.93 g/cm^3^), and *r* is the average cell radius.

As presented in Table 2, increasing charcoal loading increases the average NRF pore size, decreasing the porosity and cell density. On the other hand, increasing silica loading decreases the average pore size, increasing the porosity and cell density. Although the density of the NRF increases with increasing filler loading, the cell density is more complicated. For charcoal loading, the density slightly increases or remains almost constant with the addition of more than 4 phr of charcoal. The cell density of the NRF with charcoal loading decreases and becomes almost constant with the addition of more than 4 phr of charcoal. Since charcoal can act as the nucleating agent, which can promote foam growth, excess charcoal may stop acting as a filler and become a nucleating agent, resulting in an almost constant density with a larger average pore size and smaller porosity and cell density. For silica loading, the density increases as increasing silica loading, indicating the decreasing of the average pore size while the cell density increases.

### 3.3. Thermodynamic Aspects

Thermodynamic studies of the deformation process in uncrosslinked rubbers have already been discussed [27,28,29]. Most of the works showed the results of the temperature dependence of the stress in the extended state. In the present work, the mechanical compression properties of the crosslinked NRF samples are remarkable, especially for the %compression set and %recovery; thus, it is interesting to investigate the thermodynamic aspects. From the perspective of thermodynamics, the elasticity of rubber attributes to the changes in the conformations of rubber molecules from the unstrained molecules to the strained molecules. Such changes are related to the changes of internal energy and entropy associated with the deformation process as the following relationship [27,30,31]:

(11) $$F\;=\;\left(\frac{\partial U}{\partial L}\right)-T\left(\frac{\partial S}{\partial L}\right)\;=\;F_{u}\;+\;F_{s}$$

(12) $$F_{u}\;=\;\left(\frac{\partial U}{\partial L}\right)\;\;$$

(13) $$F_{s}\;=\;-T\left(\frac{\partial S}{\partial L}\right)\;\;$$

where *F* is the compression force causing changes in the length of NRF (*L*), *U* is the internal energy of NRF, *T* is the temperature used in the experiment, and *S* is the entropy of NRF. When plotting the compression force graph as a function of the conducted temperature, the interception at 0 K is equal to *F*_u_, and the slope multiplied by the temperature is equal to *F*_s_.

**Figure 7:**
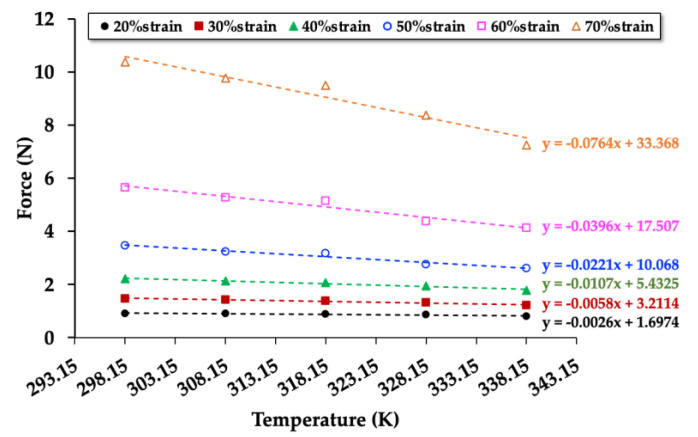
Force at a constant strain as a function of the temperature of the control NRF with a minimum of R^2^ = 0.9 in each strain.

**Figure 8:**
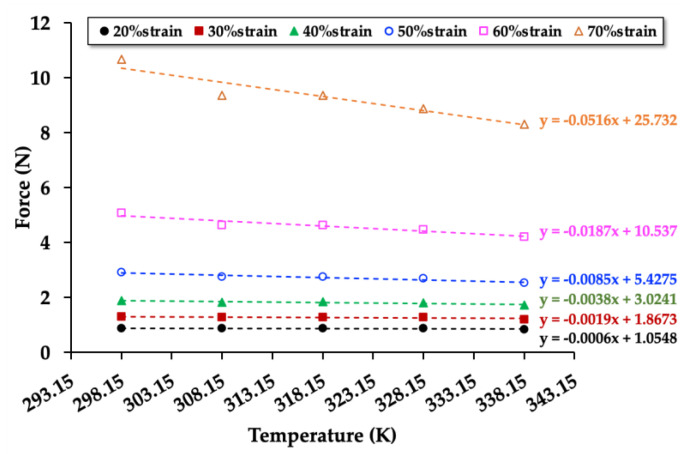
Force at a constant strain as a function of the temperature of the NRF with 8 phr of charcoal (NRF/8 Ch) with a minimum of R^2^ = 0.9 in each strain.

**Figure 9:**
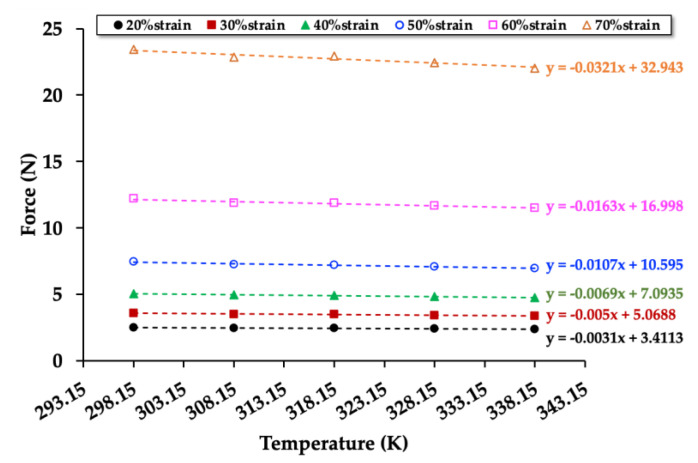
Force at a constant strain as a function of the temperature of the NRF with 8 phr of silica (NRF/8 Si) with a minimum of R^2^ = 0.9 in each strain.

To investigate the relationships between force (compression mode) and temperature, the NRF samples were compressed up to 20%strain, 30%strain, 40%strain, 50%strain, 60%strain, and 70%strain in the temperature controller chamber (at 298.15, 308.15, 318.15, 328.15, and 338.15 K). The relationships between compression force and temperature of the control NRF, NRF with 8 phr of charcoal, and NRF with 8 phr of silica are presented in Figure 7, Figure 8 and Figure 9, respectively. The graphs of the other samples are presented in Figures S1–S6. The results reveal that the compression force to the sample increases with increasing %strain. At a given strain, the compression force decreases with increasing temperature. Moreover, the slope decreases at a higher strain due to a decrease of the rubber chains’ degree of freedom in the NRF, which is unfavorable for entropy.

Figure 7, Figure 8 and Figure 9 show the values of the compression force (*F*) and relative internal energy contributing to the compression force (*F*_u_/*F*) at 298.15 K can be calculated as indicated in Table 3. The results of the other samples are presented in Table S1. The values of *F*_u_ and *F* increase with increasing compression strain, whereas the values of *F*_u_/*F* decrease. Since the internal energy (*U*) is varied by the compression force (*F*), the internal energy increases with increasing compression force. The entropy (*S*) can be varied by the length of the NRF, indicating the degree of freedom of rubber chains during the compression process. The compression causes a reduction in the length of the NRF, leading to a decrease in the rubber chains’ degree of freedom. Thus, the entropy of compressed NRF is also reduced.

**Figure 10:**
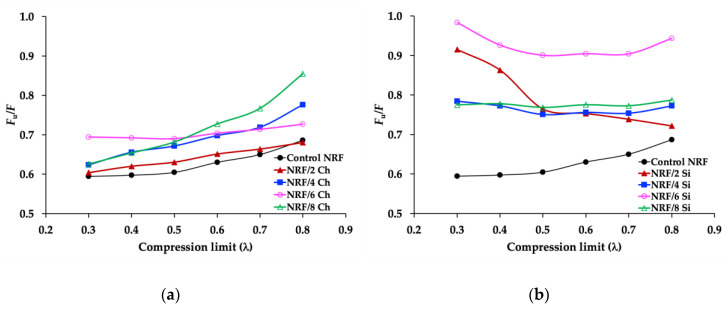
Relative internal energy contribution to the compression force (*F*_u_/*F*): (**a**) NRF with charcoal loading compared to control NRF; (**b**) NRF with silica loading compared to control NRF.

The *F*_u_/*F* values of uncrosslinked rubber in the extension mode are typically in the range of 0.1–0.2 [27]. In this work, the *F*_u_/*F* values of the crosslinked NRF in the compression mode are in the range of 0.6–0.8, which are approximately three times higher than those of the literature review. The difference in *F*_u_/*F* values could come from the material structures (uncrosslinked rubber vs. crosslinked rubbers) and the test methods (extension mode vs. compression mode). The NRFs with fillers have higher *F*_u_/*F* values than the control NRF at a given strain level, possibly explained by the interaction of rubber and filler, which promotes changes of entropy in the deformation process. The slope direction of the *F*_u_/*F* values of the NRF with charcoal and NRF with silica are different (Figure 10). This is due to the control NRF and NRF with charcoal loading possess different degrees of freedom of rubber chains at different compression limits, and lower compression limit leads to lower *F*_u_/*F* values. However, the NRFs with silica loading possess a similar degree of freedom of rubber chains at different compression limits. This indicates that the stability of the degree of freedom of rubber chains at different compression strains or compression limits (λ) is related to the high mechanical property of the NRF with silica loading. Although the compression strength of the NRF with filler increased with the increment of filler, the ratio of *F*_u_/*F* indicates that the addition of filler affects the mechanical properties in the aspect of thermodynamics.

We also investigated the change in Gibbs free energy (Δ*G*) and entropy (Δ*S*) in the NRF with fillers compared to the control NRF. These thermodynamic parameters can be calculated by the Flory–Huggins equation and statistical theory of rubber elasticity as follows [17,32]:

(14) $$∆G=\;RT\left[\ln\left(1-V_{r}\right)+V_{r}+V_{r}^{2}\chi \right]$$

(15) $$\;\;∆S=\;-\frac{∆G}{T}\;\;$$

where *R* is the gas constant (8.3145 J/mol·K), and *T* is the test temperature (300.15 K).

From the perspective of the crosslinking density, Δ*G* and Δ*S* are shown in Table 4. The volume fraction of rubber (*V*_r_) with fillers is higher than the control NRF. The swelling behavior of the NRF with various filler loadings decreases with increasing filler loading, indicating that the filler enhances the rubber swelling resistance against the penetration of the solvent. Since the filler is the hard phase, which is impermeable to solvent molecules, there must be a higher interaction between phases and more rubber chains attached to the filler surface. Hence, the swelling ability of NR is reduced while increasing the volume fraction of rubber [17].

Table 4 shows that all samples present a negative Δ*G*, which decreases with increasing filler and is a favorable spontaneous system. This is due to the restriction in the ability of the rubber chain motion in the presence of filler, resulting in a decrease in the Gibbs free energy, which can be attributed to good compatibility between polymer and filler [17,32]. Δ*S* increases with increasing filler loading, which is favorable in thermodynamics. This result is in good agreement with the result of the *F*_u_/*F* value.

**Figure 11:**
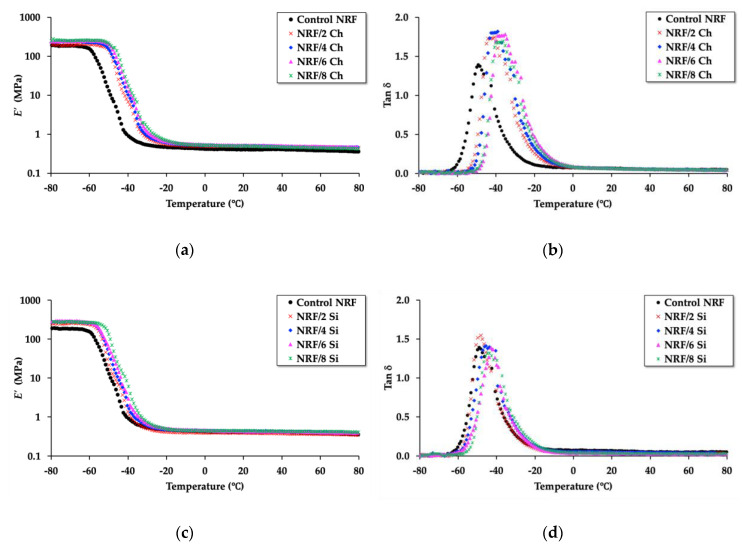
Storage modulus (*E’*) and tan δ as a function of the temperature of the NRF with various fillers: (**a**) storage modulus of the NRF with charcoal loading compared to the control NRF; (**b**) tan δ of the NRF with charcoal loading compared to the control NRF; (**c**) storage modulus of the NRF with silica loading compared to the control NRF; (**d**) tan δ of the NRF with silica loading compared to the control NRF.

Based on the dynamic mechanical properties of the sample, the storage modulus (*E’*) and tan δ results of the NRF with filler loading were determined by temperature sweep using dynamic mechanical analysis or DMA. The results are presented in Figure 11. The addition of filler, both charcoal and silica, affects the storage modulus and tan δ.

**Figure 12:**
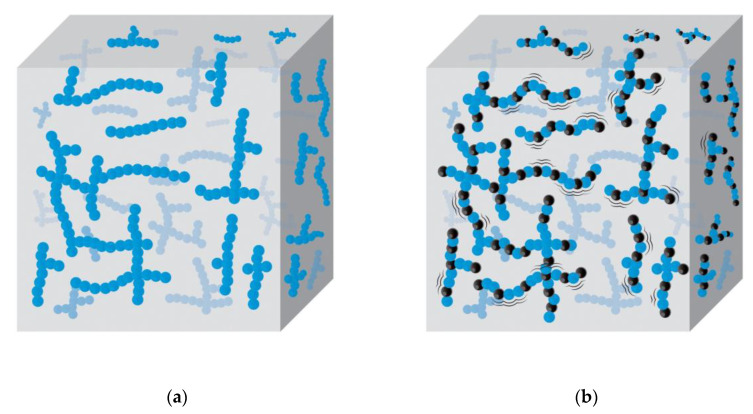
Model of the NRF with rubber chains (blue spot) and fillers (black spot): (**a**) control NRF; (**b**) NRF with filler, which increases the volume fraction of filler (∆*V*_f_) induces more stress relaxation (∆*S*t), resulting in a more pronounced entropy (∆*S*).

The DMA results presented in Table 5 reveal that the storage modulus in both the glassy state and rubbery state of the NRF with filler loading is higher than the control NRF. The addition of filler decreases the free volume within the foam, which causes more rigidity, resulting in a higher storage modulus in the glassy state [33]. The storage modulus in the rubbery state of the control NRF is lower than the NRF with filler loading. This is due to the effect of the filler on the relaxation time of rubber chains. Increasing filler loading increases the volume fraction of filler (∆*V*_f_), which causes a higher stress relaxation rate of rubber molecules where they require more time to unload the applied force [17,33]. This affects the degree of freedom of the rubber molecules to be more pronounced, i.e., when there is a greater number of interactions between the rubber chains and filler, the stress relaxation rate is increased, resulting in an increase in entropy [33,34]. Therefore, we can propose a model of the control NRF (Figure 12a) compared to the NRF with filler loading (Figure 12b). The thermodynamic meaning of this work can be explained as follows: the change in entropy (∆*S*) of the NRF with filler loading is more pronounced compared to the control NRF due to the stress relaxation rate of rubber chains from the amplification of chain deformation between the rubber and filler interaction, as shown in Equation (16).

(16) $$∆S=\;\frac{∆V_{f}\cdot ∆St}{T}$$

where ∆*S* is the change of entropy, ∆*V*_f_ is the change of volume fraction of filler, ∆*S*t is the change of stress relaxation rate of rubber molecules, and *T* is the temperature.

Table 5 also presents the *T*_g_ value or peak of tan δ of the NRF with various fillers. The addition of filler causes this value to shift toward higher temperatures when compared to the control NRF. The shift of the *T*_g_ value toward the higher temperatures indicates ionic and hydrogen bonding interactions between the rubber chains and filler [17]. However, the nonpolar charcoal might not disperse well in the concentrated natural latex or agglomerate and, instead, form filler–filler networks within the foam. This may cause a synergy effect where the filler–filler networks might defeat the movement of the free chains of rubber. Therefore, the addition of charcoal affects higher hysteresis with increasing tan δ max and *t*_A_, resulting in lower activation enthalpy (∆*H*_a_) than the control NRF.

At the same time, NRF is well-reinforced with silica. The rubber chains within the NRF with silica are hindered to freely move, and there are interactions between rubber–filler within the NRF. Thus, it has a higher activation enthalpy than the control NRF. The tan δ max of the NRF with filler has a higher value than control NRF refers to more dissipation energy of the NRF in the existence of filler. The values of ∆*H*_a_ in this work are in the same order as in the works of Sadeghi Ghari and Jalali-Arani [17].

## 4. Conclusions

In this work, natural rubber foam (NRF) was prepared in two conditions: NRF with charcoal loading and NRF with silica loading. The results showed that increasing filler loading increases the density and mechanical properties of rubber foam. Since rubber chains may be occluded in the voids of filler causing the expansion of rubber chains, leading to increasing crosslinking density, somehow, at the same loading of vulcanizing chemicals, increasing filler loading is affected less in the crosslinking density.

Increasing filler loading increases the compression stress of NRF. The compression strength of the NRF with silica loading is higher than NFR with charcoal loading due to the better interaction within the foam structure caused by the smaller silica size, which presents a more specific surface area compared to charcoal filler. Since charcoal can act as a nucleating agent and promote foam growth, the excess of charcoal might change from being filler and become the nucleating agent, resulting in a larger average pore size and smaller porosity and cell density. Increased silica loading results in a decrease in the average pore size while the cell density increases. Thermodynamic aspects showed the relationships between the force and temperature of the NRF samples. The compression force (*F*) and internal energy force (*F*_u_) values of the NRF samples increase with increasing compression strain. The NRF with various fillers has higher *F*_u_/*F* values than the control NRF. The slope direction of the *F*_u_/*F* value of the NRF with charcoal and NRF with silica are different, which comes from the different degrees of freedom of rubber chains in the NRF samples.

The swelling behavior of the NRF with filler loading decreases with increasing filler loading compared to the control NRF. The Δ*G* decreases while Δ*S* increases with increasing filler loading, which demonstrates a favorable thermodynamic system. The ∆*H*_a_ of the control NRF is lower than NRF with silica due to the movement limitation of rubber chains, whereas NRF filled with charcoal is more complicated. Increasing filler loading increases the volume fraction of filler, causing a higher stress relaxation rate due to the attempt to relax itself of molecules; therefore, the entropy is more pronounced. All the results from the theory to applications indicate that NRF, which normally behaves like a shape-memory material, can be developed into an anti-shape-memory material by the addition of silica loading, which is favorable in thermodynamics.

## Figures and Tables

**Table 1:** Composition of raw materials for the natural rubber foam (NRF) used in this study.

Sample Name	Control NRF	NRF/ 2 Ch	NRF/ 4 Ch	NRF/ 6 Ch	NRF/ 8 Ch	NRF/ 2 Si	NRF/ 4 Si	NRF/ 6 Si	NRF/ 8 Si
Chemicals	(phr ^1^)								
NRL	100	100	100	100	100	100	100	100	100
KO	3.63	3.63	3.63	3.63	3.63	3.63	3.63	3.63	3.63
Vulcanizing chemicals	4.00	4.00	4.00	4.00	4.00	4.00	4.00	4.00	4.00
WingL	1.00	1.00	1.00	1.00	1.00	1.00	1.00	1.00	1.00
ZnO	2.80	2.80	2.80	2.80	2.80	2.80	2.80	2.80	2.80
DPG	0.67	0.67	0.67	0.67	0.67	0.67	0.67	0.67	0.67
SSF	1.66	1.66	1.66	1.66	1.66	1.66	1.66	1.66	1.66
Charcoal	-	2.00	4.00	6.00	8.00	-	-	-	-
Silica	-	-	-	-	-	2.00	4.00	6.00	8.00

^1^ Parts per hundred of rubber.

**Table 2:** Average pore size, porosity, and cell density of the control NRF and NRF with filler loading.

Sample Name	Average Pore Size (±0.300 mm)	Porosity (±1.00%)	Cell Density (±150 cm^−3^)
Control NRF	0.836	31.39	3041
NRF/2 Ch	0.860	31.69	2778
NRF/4 Ch	0.988	27.58	1807
NRF/6 Ch	1.081	25.46	1379
NRF/8 Ch	1.092	25.04	1333
NRF/2 Si	1.079	28.57	1412
NRF/4 Si	0.909	28.09	2316
NRF/6 Si	0.876	27.49	2546
NRF/8 Si	0.673	26.34	5606

**Table 3:** Compression strain, compression limit, *F*_u_, *F*, and *F*_u_/*F* values of the control NRF and NRF with various fillers at 298.15 K.

Sample Name	Compression Strain (%)	Compression Limit (λ)	*F*_u_ (N)	*F* (N)	*F*_u_/*F*
Control NRF	20	0.8	1.70	2.47	0.6865
	30	0.7	3.21	4.94	0.6500
	40	0.6	5.43	8.62	0.6300
	50	0.5	10.07	16.66	0.6044
	60	0.4	17.51	29.31	0.5972
	70	0.3	33.37	56.15	0.5943
NRF/8 Ch	20	0.8	1.05	1.23	0.8550
	30	0.7	1.87	2.43	0.7672
	40	0.6	3.02	4.16	0.7275
	50	0.5	5.43	7.96	0.6817
	60	0.4	10.54	16.11	0.6540
	70	0.3	25.73	41.12	0.6258
NRF/8 Si	20	0.8	3.41	4.34	0.7868
	30	0.7	5.07	6.56	0.7727
	40	0.6	7.09	9.15	0.7752
	50	0.5	10.60	13.79	0.7686
	60	0.4	17.00	21.86	0.7777
	70	0.3	32.94	42.51	0.7749

**Table 4:** Calculated parameters from crosslinking density results for the control NRF and NRF with various fillers.

Sample Name	Swelling Ratio	Volume Fraction of Rubber (*V*_r_)	Δ*G* (J/mol)	Δ*S* (J/mol·K)
Control NRF	2.83	0.2377	−29.15	0.0971
NRF/2 Ch	2.33	0.2755	−42.85	0.1428
NRF/4 Ch	2.24	0.2823	−45.74	0.1524
NRF/6 Ch	2.25	0.2790	−44.33	0.1477
NRF/8 Ch	2.23	0.2843	−46.61	0.1553
NRF/2 Si	2.33	0.2739	−42.21	0.1406
NRF/4 Si	2.30	0.2732	−41.91	0.1396
NRF/6 Si	2.24	0.2777	−43.77	0.1458
NRF/8 Si	2.19	0.2839	−46.41	0.1546

**Table 5:** Obtained parameters by the dynamic mechanical analysis (DMA) test for the control NRF and NRF with various fillers.

Sample Name	*E*_g_ @ −70 °C (MPa)	*E*_r_ @ 0 °C (MPa)	*T*_g_ (°C)	tan δ max	*t*_A_	(∆*H*_a_)_avg_ (kJ·K/mol)
Control NRF	184.05	0.42	−49.08	1.39	31.08	128.33
NRF/2 Ch	193.35	0.49	−40.75	1.75	39.04	107.81
NRF/4 Ch	248.15	0.51	−37.17	1.82	40.87	109.92
NRF/6 Ch	240.76	0.54	−35.17	1.78	40.24	112.16
NRF/8 Ch	251.01	0.52	−36.17	1.69	38.13	118.76
NRF/2 Si	250.74	0.39	−48.17	1.54	28.25	151.17
NRF/4 Si	283.10	0.44	−45.50	1.41	30.51	143.53
NRF/6 Si	294.05	0.45	−43.00	1.39	25.73	174.06
NRF/8 Si	280.63	0.44	−44.67	1.32	27.60	159.44

## Supplementary Materials

The following are available online at https://www.mdpi.com/2073-4360/12/11/2745/s1, Figure S1. Force at a constant strain as a function of the temperature of the NRF with 2 phr of charcoal (NRF/2 Ch) with a minimum of R^2^ = 0.9 in each strain. Figure S2. Force at a constant strain as a function of the temperature of the NRF with 4 phr of charcoal (NRF/4 Ch) with a minimum of R^2^ = 0.9 in each strain. Figure S3. Force at a constant strain as a function of the temperature of the NRF with 6 phr of charcoal (NRF/6 Ch) with a minimum of R^2^ = 0.9 in each strain. Figure S4. Force at a constant strain as a function of the temperature of the NRF with 2 phr of silica (NRF/2 Si) with a minimum of R^2^ = 0.9 in each strain. Figure S5. Force at a constant strain as a function of the temperature of the NRF with 4 phr of silica (NRF/4 Si) with a minimum of R^2^ = 0.9 in each strain. Figure S6. Force at a constant strain as a function of the temperature of the NRF with 6 hr of silica (NRF/6 Si) with a minimum of R^2^ = 0.9 in each strain. Table S1. Compression strain, compression limit, *F*_u_, *F*, and *F*_u_/*F* values of the control NRF and NRF with various fillers at 298.15 K.
