# Supplementary material for: Effect of Fillers on the Recovery of Rubber Foam: From Theory to Applications
Source: Polymers (Basel). 2020 Nov 19;12(11):2745. doi: 10.3390/polym12112745 (PMC7699531; doi:10.3390/polym12112745)
Supplement: Supplementary file 1 [file polymers-12-02745-s001.pdf]

Supplementary Materials

Effect of fillers on recovery of rubber foam: From theory to applications

Thridsawan Prasopdee [1] and Wirasak Smitthipong [1,2,3,*]

[1] Specialized center of Rubber and Polymer Materials in agriculture and industry (RPM), Department of Materials Science, Faculty of Science, Kasetsart University, Chatuchak, Bangkok 10900, Thailand; thridsawan@gmail.com
[2] Office of research integration on target-based natural rubber, National Research Council of Thailand (NRCT), Chatuchak, Bangkok 10900, Thailand.
[3] Office of natural rubber research program, Thailand Science Research and Innovation (TSRI), Chatuchak, Bangkok 10900, Thailand.
* Correspondence: fsciwssm@ku.ac.th

Received: date; Accepted: date; Published: 19 November 2020

Keywords: rubber foam; filler; charcoal; silica; compression set; recovery; thermodynamics

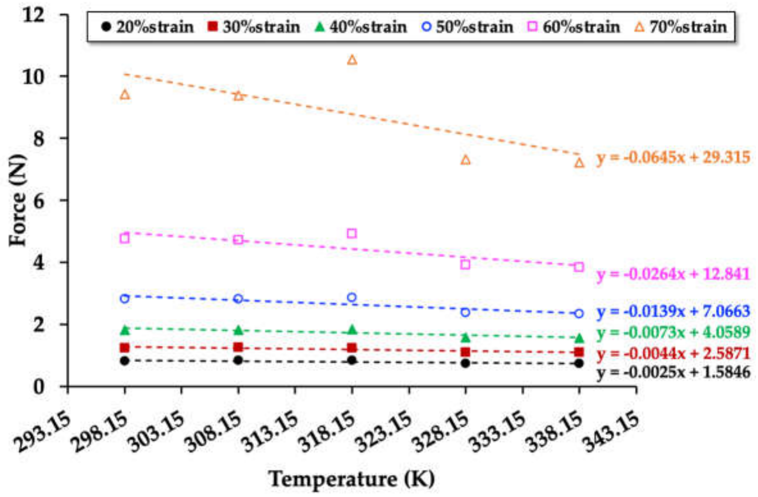

Figure S1. Force at constant strain as a function of temperature of NRF with charcoal 2 phr (NRF/2 Ch) which minimum of $R^2 = 0.9$ in each strain.

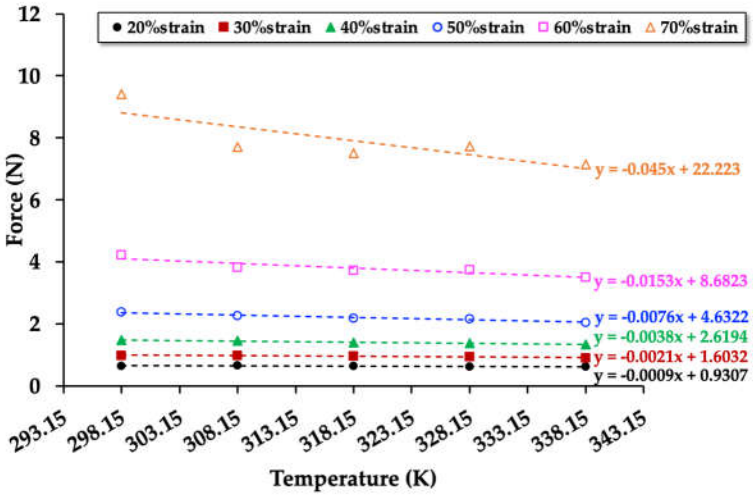

Figure S2. Force at constant strain as a function of temperature of NRF with charcoal 4 phr (NRF/4 Ch) which minimum of R^2 = 0.9 in each strain.

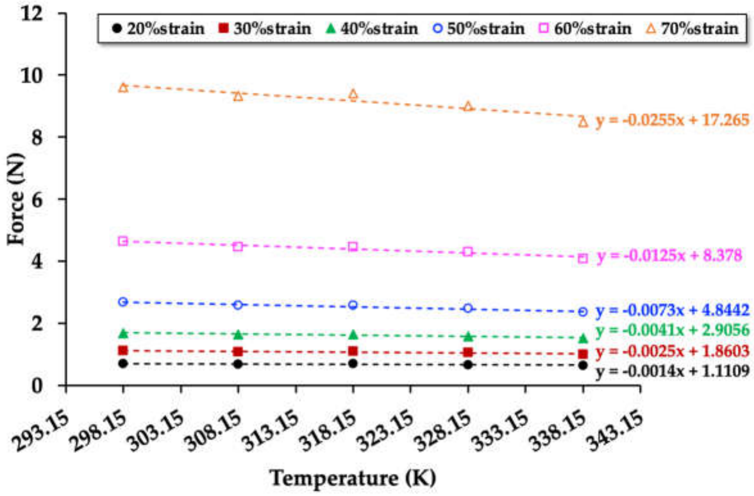

Figure S3. Force at constant strain as a function of temperature of NRF with charcoal 6 phr (NRF/6 Ch) which minimum of R^2 = 0.9 in each strain.

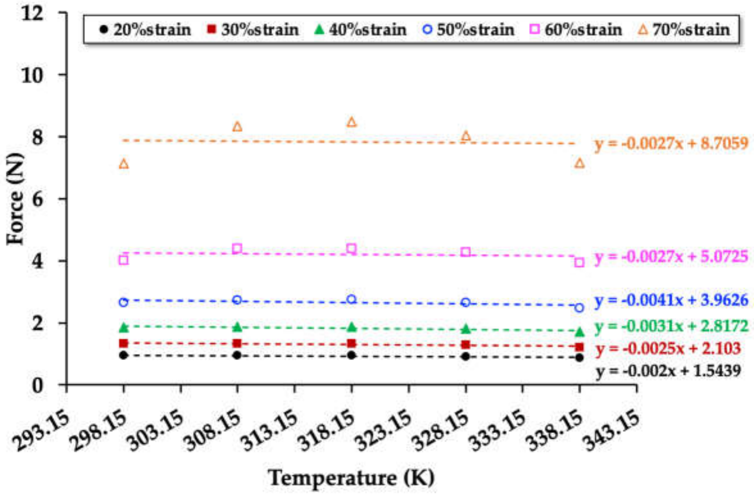

Figure S4. Force at constant strain as a function of temperature of NRF with silica 2 phr (NRF/2 Si) which minimum of R^2 = 0.9 in each strain.

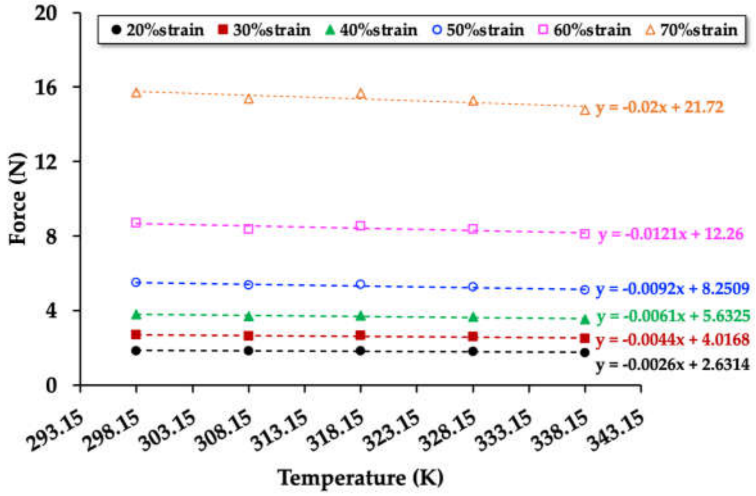

Figure S5. Force at constant strain as a function of temperature of NRF with silica 4 phr (NRF/4 Si) which minimum of R^2 = 0.9 in each strain.

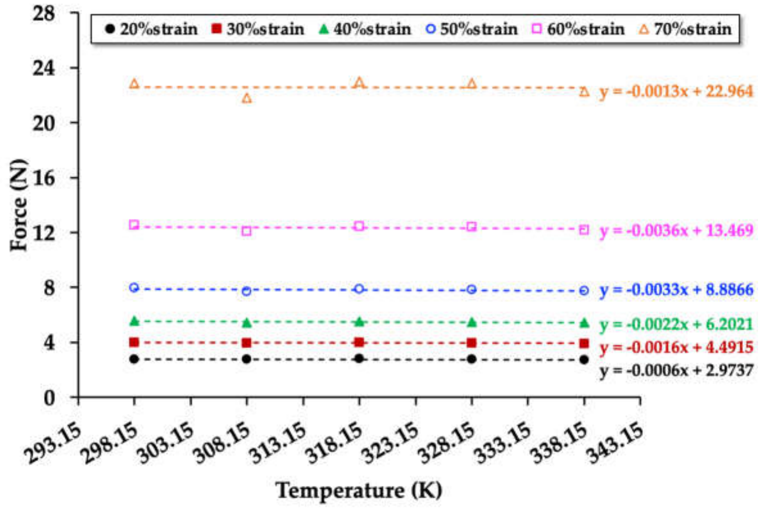

Figure S6. Force at constant strain as a function of temperature of NRF with silica 6 phr (NRF/6 Si) which minimum of R^2 = 0.9 in each strain.

Table S1. Compression strain, compression limit, F_u, F, and Fu/F value of control NRF and NRF with filler loading at 298.15 K.

Sample Name	Compression strain (%)	Compression limit (λ)	F_u (N)	F (N)	F_u/F
Control NRF	20	0.8	1.70	2.47	0.6865
	30	0.7	3.21	4.94	0.6500
	40	0.6	5.43	8.62	0.6300
	50	0.5	10.07	16.66	0.6044
	60	0.4	17.51	29.31	0.5972
	70	0.3	33.37	56.15	0.5943
NRF/2 Ch	20	0.8	1.58	2.33	0.6801

Sample Name	Compression strain (%)	Compression limit (λ)	F_u (N)	F (N)	F_u/F
	30	0.7	2.59	3.90	0.6635
	40	0.6	4.06	6.24	0.6509
	50	0.5	7.07	11.21	0.6303
	60	0.4	12.84	20.71	0.6200
	70	0.3	29.32	48.55	0.6039
NRF/4 Ch	20	0.8	0.93	1.20	0.7762
	30	0.7	1.60	2.23	0.7191
	40	0.6	2.62	3.75	0.6981
	50	0.5	4.63	6.90	0.6715
	60	0.4	8.68	13.24	0.6556
	70	0.3	22.22	35.64	0.6235
NRF/6 Ch	20	0.8	1.11	1.53	0.7269
	30	0.7	1.86	2.61	0.7139
	40	0.6	2.91	4.13	0.7039
	50	0.5	4.84	7.02	0.6900
	60	0.4	8.38	12.10	0.6921
	70	0.3	17.27	24.87	0.6943
NRF/8 Ch	20	0.8	1.05	1.23	0.8550
	30	0.7	1.87	2.43	0.7672
	40	0.6	3.02	4.16	0.7275
	50	0.5	5.43	7.96	0.6817
	60	0.4	10.54	16.11	0.6540
	70	0.3	25.73	41.12	0.6258
NRF/2 Si	20	0.8	1.54	2.14	0.7214
	30	0.7	2.10	2.85	0.7383
	40	0.6	2.82	3.74	0.7530
	50	0.5	3.96	5.19	0.7642
	60	0.4	5.07	5.88	0.8630
	70	0.3	8.71	9.51	0.9154
NRF/4 Si	20	0.8	2.63	3.41	0.7724
	30	0.7	4.02	5.33	0.7538
	40	0.6	5.63	7.45	0.7559
	50	0.5	8.25	10.99	0.7505

**Sample Name**	**Compression strain (%)**	**Compression limit (λ)**	F_u **(N)**	F **(N)**	F_u/F
	60	0.4	12.26	15.87	0.7726
	70	0.3	21.72	27.68	0.7846
NRF/6 Si	20	0.8	2.97	3.15	0.9433
	30	0.7	4.49	4.97	0.9040
	40	0.6	6.20	6.86	0.9044
	50	0.5	8.89	9.87	0.9003
	60	0.4	13.47	14.54	0.9262
	70	0.3	22.96	23.35	0.9834
NRF/8 Si	20	0.8	3.41	4.34	0.7868
	30	0.7	5.07	6.56	0.7727
	40	0.6	7.09	9.15	0.7752
	50	0.5	10.60	13.79	0.7686
	60	0.4	17.00	21.86	0.7777
	70	0.3	32.94	42.51	0.7749

Publisher's Note: MDPI stays neutral with regard to jurisdictional claims in published maps and institutional affiliations.

© 2020 by the authors. Licensee MDPI, Basel, Switzerland. This article is an open access article distributed under the terms and conditions of the Creative Commons Attribution (CC BY) license (http://creativecommons.org/licenses/by/4.0/).
